# Negative relationship between dry matter intake and the temperature-humidity index with increasing heat stress in cattle: a global meta-analysis

**DOI:** 10.1007/s00484-021-02167-0

**Published:** 2021-07-20

**Authors:** J. Chang-Fung-Martel, M. T. Harrison, J. N. Brown, R. Rawnsley, A. P. Smith, H. Meinke

**Affiliations:** 1 Tasmanian Institute of Agriculture, University of Tasmania, Sandy Bay, TAS 7001 Australia; 2 School of Life and Environmental Sciences, The University of Sydney, Camden, NSW 2570 Australia; 3 Tasmanian Institute of Agriculture, University of Tasmania, Burnie, TAS 7320 Australia; 4 CSIRO Oceans and Atmosphere, Castray Esplanade, Battery Point, TAS 7001 Australia; 5 ICRISAT, Patancheru, 502 324 Telangana India; 6 University of Tasmania, Hobart, TAS 7001 Australia

**Keywords:** Dairy, Adaptation, Impact, Hyperthermia, Temperature, Milk production

## Abstract

Changes in frequency and severity of heat waves due to climate change pose a considerable challenge to livestock production systems. Although it is well known that heat stress reduces feed intake in cattle, effects of heat stress vary between animal genotypes and climatic conditions and are context specific. To derive a generic global prediction that accounts for the effects of heat stress across genotypes, management and environments, we conducted a systematic literature review and a meta-analysis to assess the relationship between dry matter intake (*DMI*) and the temperature-humidity index (*THI*), two reliable variables for the measurement of feed intake and heat stress in cattle, respectively. We analysed this relationship accounting for covariation in countries, breeds, lactation stage and parity, as well as the efficacy of various physical cooling interventions. Our findings show a significant negative correlation (*r* =  − 0.82) between *THI* and *DMI*, with *DMI* reduced by 0.45 kg/day for every unit increase in *THI*. Although differences in the *DMI*-*THI* relationship between lactating and non-lactating cows were not significant, effects of *THI* on *DMI* varied between lactation stages. Physical cooling interventions (e.g. provision of animal shade or shelter) significantly alleviated heat stress and became increasingly important after *THI* 68, suggesting that this *THI* value could be viewed as a threshold for which cooling should be provided. Passive cooling (shading) was more effective at alleviating heat stress compared with active cooling interventions (sprinklers). Our results provide a high-level global equation for *THI-DMI* across studies, allowing next-users to predict effects of heat stress across environments and animal genotypes.

## Introduction

Excessive heat stress in cattle (subfamily Bovinae) has been associated with reduced productivity and profitability (Chang-Fung-Martel et al. 2017; Harrison et al. 2017), with heat events in the USA associated with losses of over one billion dollars in 2006 (Collier and Burgos-Zimbelmanm 2007). Cattle heat stress can result in factors including (i) reduced feed intake leading to impaired body weight gains and milk production, (ii) reduced fertility rates and reproductive performance, (iii) increased production costs associated with cooling and other heat mitigation strategies and (iv) increased mortalities. Physical responses to heat in cattle include increased body temperature and respiratory rate, panting, increased water intake and reduced dry matter intake (*DMI*) (Magdub et al. 1982; Wise et al. 1988). These responses trigger physiological mechanisms to increase heat evaporation, dissipate internal heat load and therefore cool down. However, failure to effectively dissipate heat results in an accumulation of internal heat load that compromises homeostasis and increase maintenance requirements by up to 32% (Eastridge et al. 1998; Fox and Tylutki 1998; National Research Council 1981).

Reduced *DMI* caused by heat stress on the one hand decreases energy and nutrient intake, but on the other hand increases energy demand. Together, these factors reduce productivity. Lower *DMI* may be attributed to (1) behavioural adaptations to ameliorate internal heat load due to feed fermentation (Ominski et al. 2002a) and (2) changes in blood distribution away from the gut, uterus, udder and internal organs to favour peripheral circulation that facilitates heat exchange with the environment (Garner, 2017). This results in depressed rumination and longer time for feed to be digested. Independent of nutrient intake, energy requirements are reprioritised (Baumgard and Rhoads 2012), resulting in shifted patterns of carbohydrate, protein and fat metabolism characterised by increased insulin levels and reduced lipolytic activation (Baumgard and Rhoads 2012). As such, the ability of heat-stressed dairy cows to mobilise adipose tissue is impaired, leaving less energy for milk production (Baumgard and Rhoads 2012; Rhoads, 2009; Wheelock et al. 2010a). While reduced milk production during warmer conditions cannot be fully attributed to reduced *DMI* alone (Gao et al., 2017), reduced *DMI* is a good indicator of heat stress onset and is known to have direct impact on productivity.

The temperature-humidity index (*THI*), a function of ambient temperature and relative humidity, is considered the most widely used climatic indicator of heat stress in dairy cattle (Chang-Fung-Martel et al. 2017; Polsky and Keyserlingk, 2017). *THI* is strongly correlated with increased heart rate, respiratory rate and rectal and vaginal temperature in animals exposed to hot environmental conditions. While there have been many studies on the relationship between *THI* and *DMI* in cattle (e.g. Ammer et al. 2018; Allen et al. 2015; Bouraoui et al. 2002; Holter et al. 1997; Holter et al. 1996; Rodriquez et al., 1985), such studies have often been conducted under site-specific conditions. This diversity of experimental treatments as well as a lack of standardisation of feed and heat stress metrics makes comparisons of results, metrics and general principles across studies difficult. Despite known relationships between *THI* and *DMI*, many modelling approaches of future climate impacts on livestock systems ostensibly have not accounted for direct effects of heat stress on animals (e.g. Harrison et al. 2016; Pembleton et al. 2016). In this study, we aimed to develop a more general relationship between *THI* and *DMI* that could be used to predict dry matter intake reduction across environments, management and animal genotypes and that could be used as basis to improve future modelling approaches. We conducted a systematic literature review and meta-analysis to analyse how heat stress impacted on *DMI*, allowing a standardised heat stress comparison across regions. Such comparison is important in global studies comparing the effects of climate change on animal production systems. As part of our review, we identified subclasses that may be relatively more impacted by heat stress and assessed the efficacy of various adaptations to promote cooling.

## Materials and methods

### Literature search and study inclusion criteria

A systematic literature review was conducted using ISI Web of Science (Clarivate Analytics, Pennsylvania, USA; https://apps.webofknowledge.com/) and Scopus (Elsevier, Amsterdam, Netherlands; https://www.scopus.com). We used the PIC (Population, Intervention, Comparator) truncated version framework (Eriksen and Frandsen 2018) of the Preferred Reporting Items for Systematic Reviews and Meta-Analysis (PRISMA) statement (Moher et al. 2009) to identify published studies. Combinations (n = 140) of the PIC search terms shown in Table 1 were used in each online database and results were recorded in a spreadsheet, including the number of records retrieved. The review focussed on publications in English and was limited to studies that reported experimental paired observations of *DMI* and *THI*. Experiments that reported climatic variables other than *THI* or feed intake measurements other than *DMI* were discarded unless the variables allowed calculation of *THI* and *DMI*, as demonstrated by the inclusion criteria used in the meta-analysis. Studies that reported *DMI* predictive equations or those determined through modelling experiments were also excluded. All search results were imported to an Endnote library (Endnote X9, Clarivate Analytics, CA). Animal cooling strategies were grouped as either passive (no use of external energy) and active (use of external energy to enable) or none.

**Table 1:** PRISMA-PIC truncated framework including the number (*n*) of search terms used to identify scientific publications for the meta-analysis, including seven inclusion criteria in relation to the search terms

Category	*n*	Search terms	Inclusion criteria
			English language
P (population)	4	Beef, cattle, cow, dairy	*Bos taurus* species (i.e. dairy or beef cattle)
I (intervention)	7	Climate, heat, heat stress, temperature AND relative humidity, temperature humidity index, temperature-humidity index, *THI*	Temperature humidity index and/or Ambient temperature AND relative humidity
C (comparator)	5	*DMI*, dry matter intake, dry-matter-intake, feed efficiency, feed intake	Dry matter intake
Total	140	All possible combinations of search terms above	Experimental trials (not predictive modelling) Paired observations between climate and intake variables

### Data extraction and statistical analysis

Data observations for studies included in the meta-analysis were collected into individual Excel spreadsheets. Where possible, data recorded also included milk production, body temperature, breed, parity, stage of lactation, cooling strategies and country of origin. In cases where data were not tabulated, graph points were extracted using a digitiser (WebPlotDigitizer, https://automeris.io/WebPlotDigitizer/) (Drevon et al. 2017).

All data analysis was conducted using R (R Core Team 2013). Heterogeneity between studies was quantified using Higgins I^2^. A random effects model was applied to award relative weights to each study in the meta-analysis. Pearson correlations (*r*) between *DMI* and measurements commonly used to quantify heat stress in cattle (daily mean *THI*, *THI*_*min*_ and *THI*_*max*_, respiratory rate (RT), vaginal temperature (VT) and respiratory rate (RR)) were assessed using Cohen’s standard where associations were represented as weak (0.10–0.29), moderate (0.30–0.49) or strong (0.50 or greater) (Cohen 1988). Multiple lines of best fit regressions were used to assess the relationship between *DMI* and *THI* within subgroups including origin of study, breed, parity, stage of lactation and when cooling strategies were used to mitigate heat stress in dairy cows. In this study, all calculations were carried out with mean daily *THI*. Differences between subgroups were assessed using ANOVA and Tukey’s test, deemed significant at the 0.05 level. Adjusted R^2^ values are shown throughout.

## Results

### Systematic literature review

**Fig. 1:**
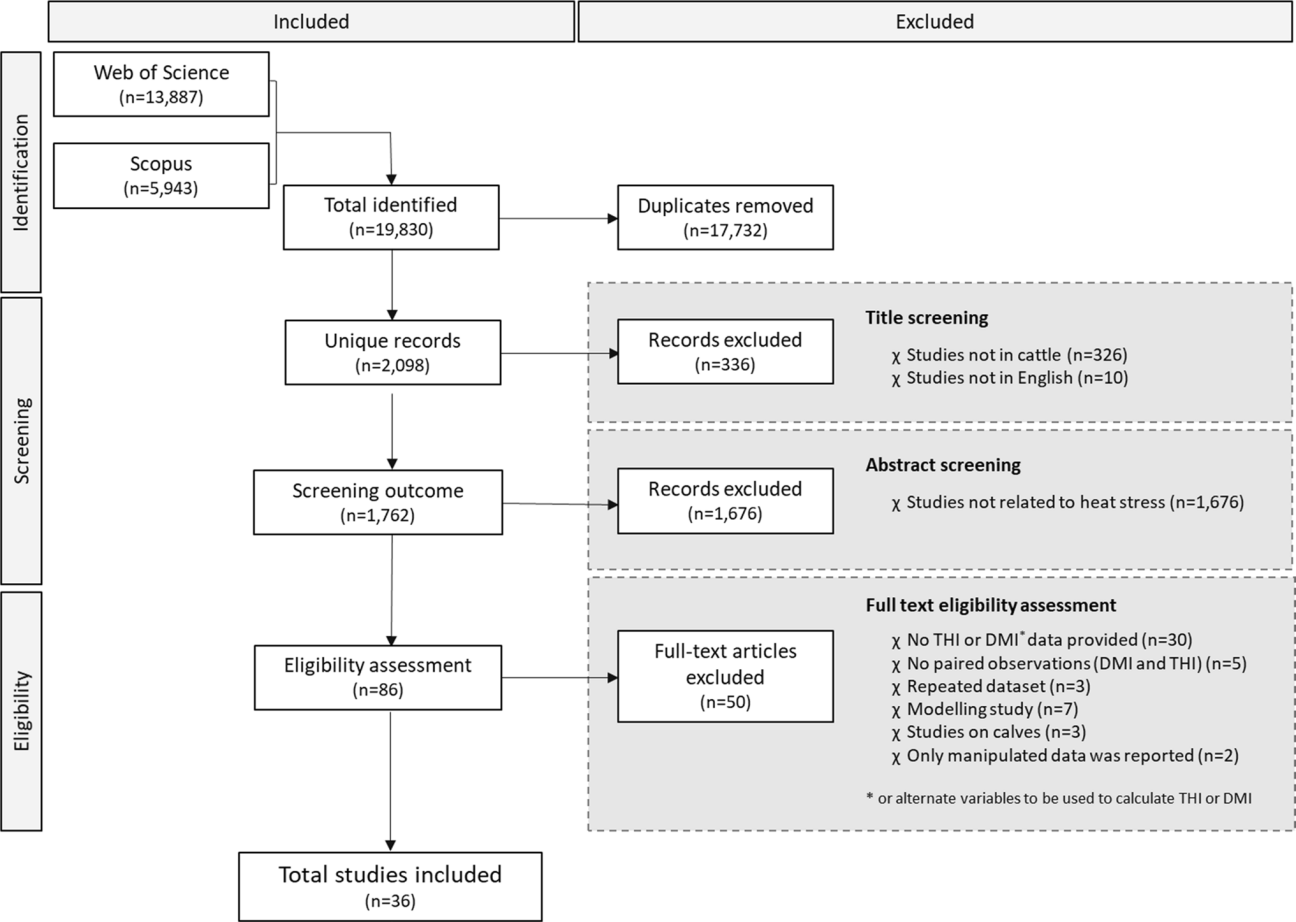
Adjusted PRISMA flow diagram describing the process undertaken to identify, screen and assess the eligibility of studies included in the meta-analysis. The number of studies (*n*) in each stage of the process outlined above is shown between parentheses

The literature search revealed 19,830 records and 2,098 unique studies. The screening process and eligibility assessment shown in Fig. 1 yielded 36 articles that met the inclusion criteria and the data extraction process yielded 676 paired observations between *THI* (mean 71.8 ± 10.4) and *DMI* (mean 15.5 ± 6.2 kg/day). Data points were derived from experiments from 15 different countries. Variables assessed included cattle breed, parity, production stage, stage of lactation, rectal temperature, respiratory rate and vaginal temperature (Table 2).

**Table 2:** Continuous (A) and discrete (B) variables included in the meta-analysis. Continuous variables show the average value and standard deviation in parenthesis

(A)	Continuous variables	Mean (± SD)
	*THI*	71.8 (± 10.4)
	*DMI* (kg/d)	15.5 (± 6.17)
	Rectal temperature (°C)	39.0 (± 0.8)
	Vaginal temperature (°C)	38.96 (± 0.7)
	Respiratory rate (breaths/min)	60.4 (± 16.9)
(B)	Discrete variables	Levels
	Country	Australia, Brazil, Canada, China, Germany, Ghana, India, Iran, Israel, Italy, Japan, South Africa, Thailand, Tunisia, USA
	Breed	Angus, Bonsmara, Charolais, Holstein–Friesian, Shorthorn, Vrindavan
	Parity	Primiparous, multiparous
	Lactation status	Lactating, non-lactating
	Stage of lactation	Early, mid and dry
	Cooling strategies	Passive, active, none

### Dry matter intake

**Fig. 2:**
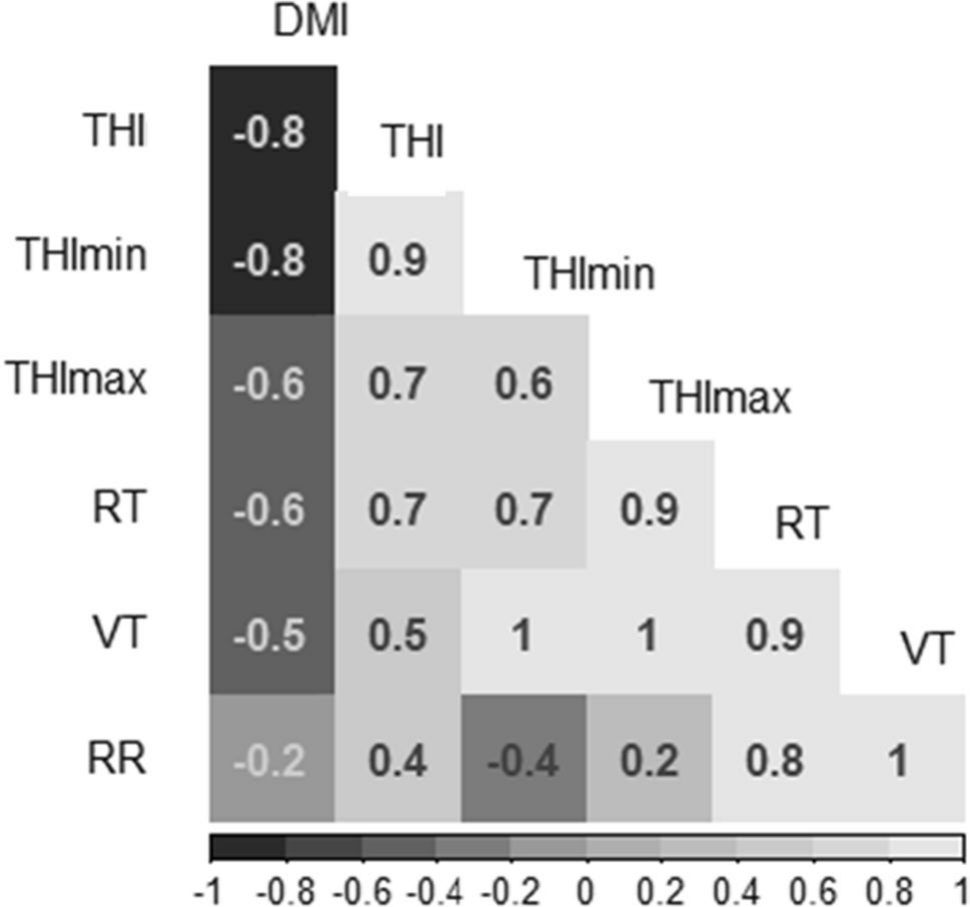
Pearson correlation coefficient matrix of dry matter intake (*DMI*), mean temperature-humidity index (*THI*), minimum temperature-humidity index (*THImin*), maximum temperature-humidity index (*THImax*), rectal temperature (RT), vaginal temperature (VT) and respiratory rate (RR). Light and dark shading represent positive and negative correlations, respectively

Relationships between *DMI* with *THI*, minimum daily *THI* (*THI*_*min*_), maximum daily *THI* (*THI*_*max*_), respiratory rate (RT), vaginal temperature (VT) and respiratory rate (RR) are shown in Fig. 2. A strong negative correlation of *r* =  − 0.82 was found between *DMI* and *THI*. *DMI* was also strongly negatively correlated with *THI* and *THI*_*min*_ and moderately correlated with *THI*_*max*_. Conversely, *DMI* was negatively correlated with *THI*, VT, RT and RR. The strongest relationships were between VT and *THI*_*max*_ or *THI*_*min*_. RR was poorly correlated with *DMI*, suggesting that feed intake does not relate well with basal respiration (Fig. 2).

**Fig. 3:**
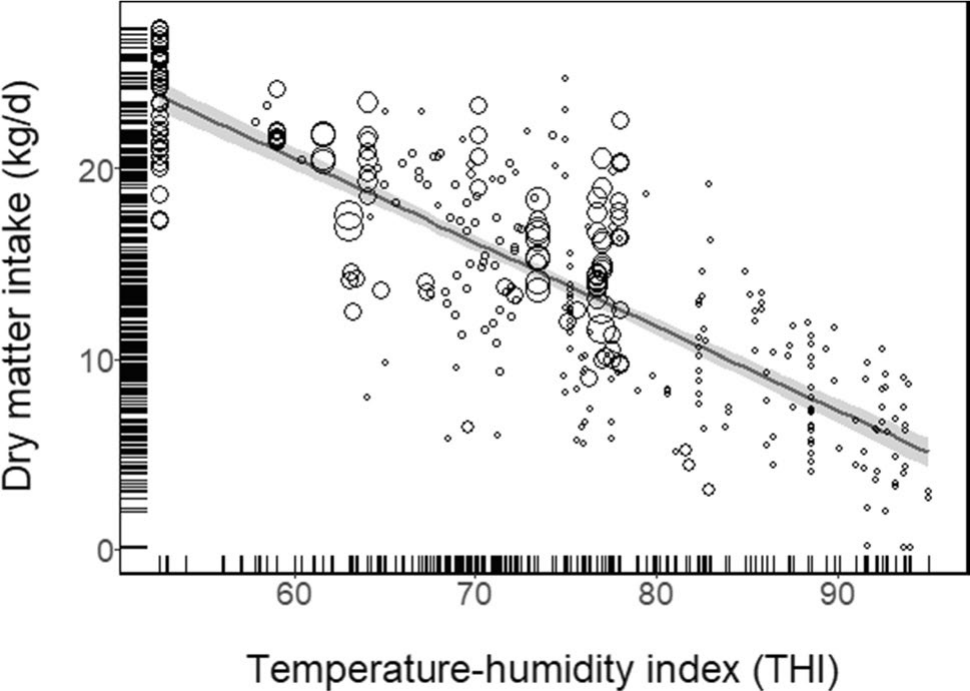
Relationship between *THI* and *DMI*, including line of best fit and 95% confidence limits (shaded grey). Rug plots show distributions of each variable on each axis. Data point sizes are proportional to weighting given to each study in the meta-analysis

We observed significant heterogeneity (I^2^ = 66.4%) between studies and applied a random effects model to assess the relationship between *THI* and *DMI*. Symbol sizes in Fig. 3 are proportional to weighting given to each study in the meta-analysis. Differences in slopes between *THI*, *THI*_min_ and *THI*_max_ were not significant (data not shown). For every unit increase in *THI*, *DMI* was reduced by 0.45 kg *DMI*/day across all datasets (*THI* = 48.29 − 0.45*DMI*; *R*^*2*^ = 0.68).

For each unit increase in *THI*, we found a reduction of 0.57 kg *DMI*/day for Asia, 0.51 kg *DMI*/day for South America, 0.48 kg *DMI*/day for Oceania, 0.42 kg *DMI*/day for Europe and 0.29 kg *DMI*/day for North America. Data points from Africa were removed from this analysis because the sample size was too small (one study and five paired observations). Changes in *DMI* at increasing *THI*s in the North American group (*n* = 73) were significantly different to that from all other continents. North American studies, encompassing data from the USA and Canada, showed the least reduction in *DMI* and Asian studies showed the largest decline in *DMI* as *THI* increased (R^2^ 0.67). The relationship between *THI*, *DMI* and milk production was not assessed due to lack of data.

### Relationships between THI and DMI within subgroups

There were large differences in the relationships between *THI* and *DMI* across subgroups. In beef cattle, *THI* (mean 77 ± 7.2) was significantly higher than in which dairy cattle were exposed (mean 72.1 ± 11.0) while beef *DMI* (mean 6.0 kg/day ± 1.7) was significantly lower than dairy *DMI* (mean 15.9 kg/day ± 6.0). Beef and dairy subgroups were statistically different with dairy (*THI* = 48.14 − 0.45*DMI*) being more impacted by heat stress than beef cattle (*THI* = 13.88 − 0.10*DMI*). Most breeds (94.5%) were Holstein–Friesian, so differences between breeds were not assessed here.

**Fig. 4:**
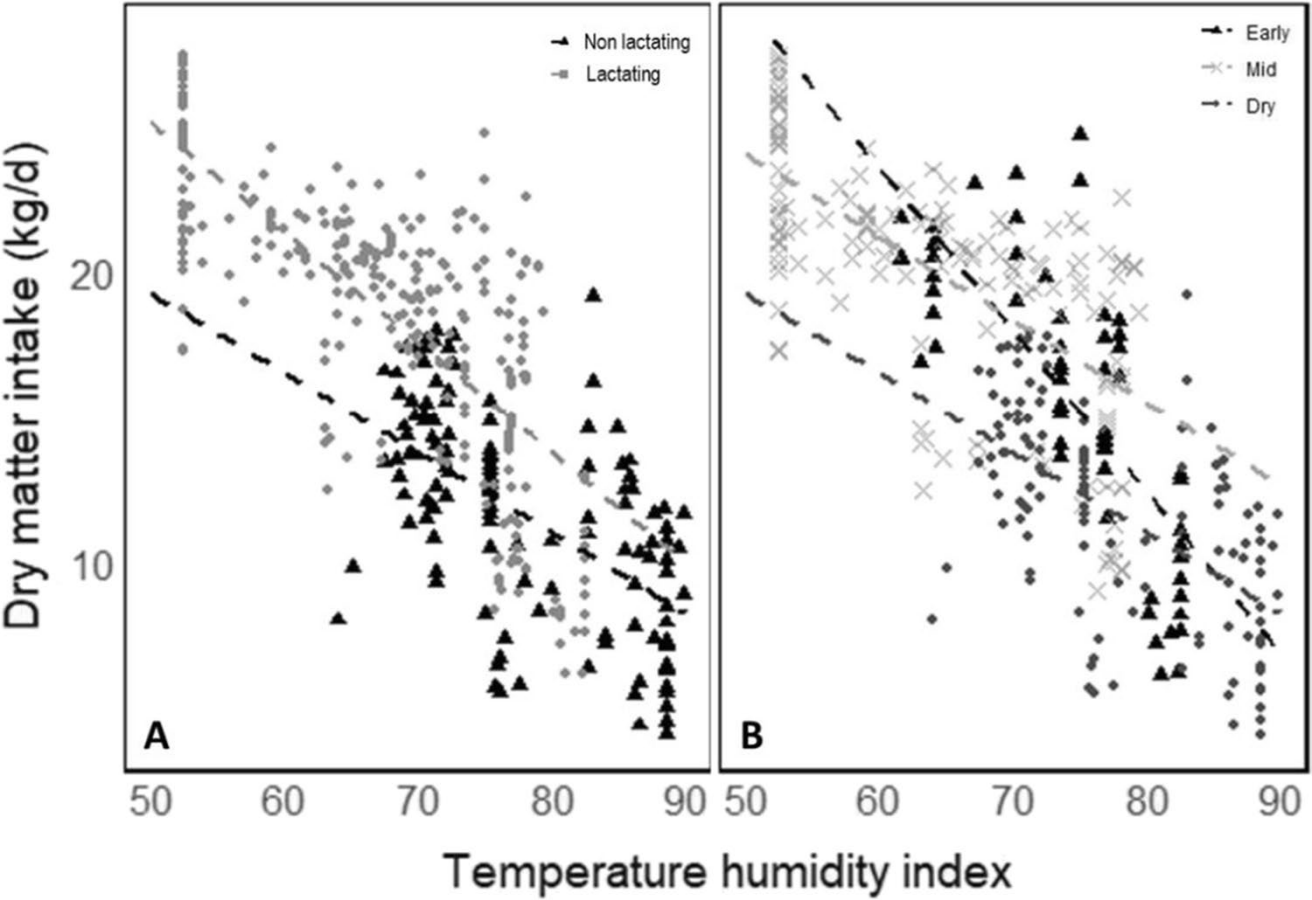
Relationships between *THI* and *DMI* segregated according to **A** lactation status (*R*^*2*^ 0.76) and **B** lactation stage (*R*^*2*^ 0.54)

For dairy cows, *THI-DMI* relationship differences between primiparous (*n* = 21) and multiparous (*n* = 398) cows were not significant. Although differences in the *THI*-*DMI* relationship between lactating (*n* = 277) and non-lactating cows (*n* = 161) were not significant (Fig. 4A), relationships were significantly different across lactation stages. Early lactation cows (*n* = 64) showed the largest reduction in *DMI* (0.56 kg *DMI*/day) per unit increase of *THI* with dry (*n* = 161) and mid (*n* = 152) lactation cows were not statistically different (Fig. 4B). *DMI* was also different between lactation stages (early 15.6 kg/day ± 4.7; mid 19.9 kg/day ± 4.2; dry 10.3 kg/day ± 4.3).

### Effects of animal cooling interventions on DMI

**Fig. 5:**
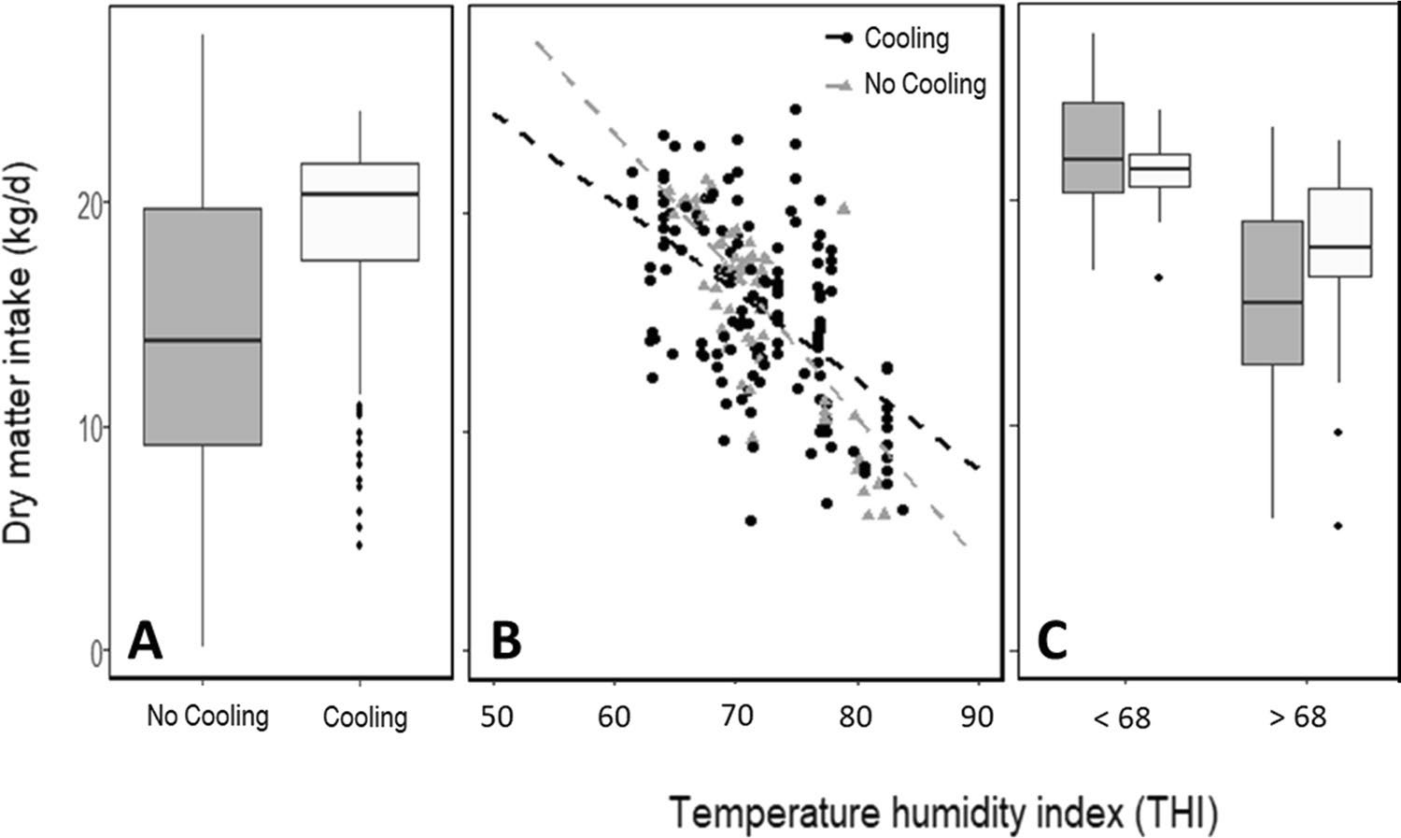
Effects of cooling interventions on *DMI*. **A** Effects of cooling interventions on the distribution of *THI* (**B**) with increasing *THI* (*R*^*2*^ 0.66) and **C** *DMI* distributions partitioned according to a *THI* threshold of 68

Thirty percent of studies examined cooling strategies. Subgroups exposed to cooling (*n* = 138) had higher *DMI* at the same *THI* (Fig. 5A) as cattle not exposed to cooling (*n* = 336). Cattle not exposed to cooling had a greater reduction in *DMI* (0.44 vs 0.36 kg *DMI*/day) per unit increase in *THI* (Fig. 5B). The cooling intervention began to take effect at *THI* 68, after which significant differences in *DMI* were observed between cooled and not cooled cattle (Fig. 5C).

**Fig. 6:**
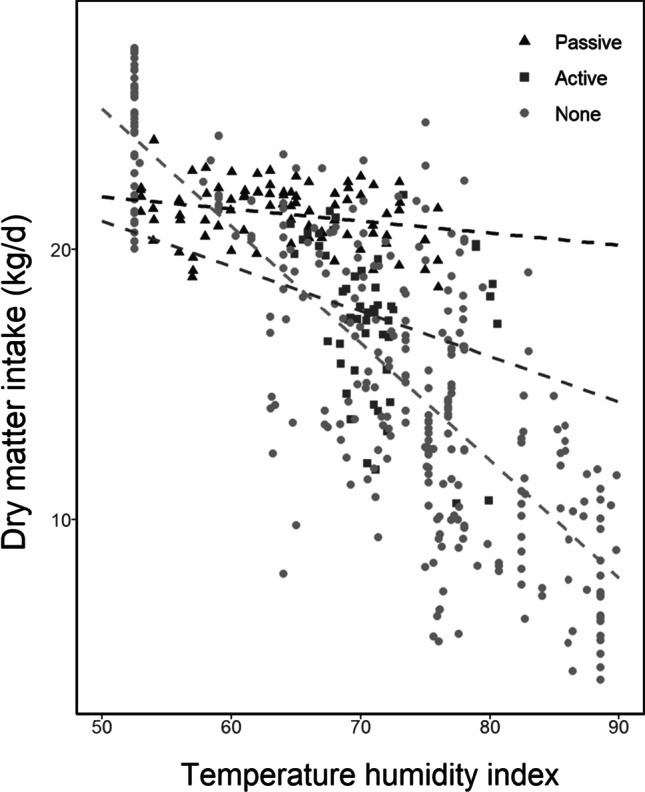
Changes in *DMI* under animal passive, active and no cooling interventions (*R*^*2*^ 0.69)

When cooling interventions were subdivided into passive cooling (e.g. shading) (*n* = 84), active cooling (e.g. fans or sprinklers) (*n* = 54) and no cooling (*n* = 336), it was shown that passive strategies were most effective at heat mitigation in *DMI* alleviation (Fig. 6). *DMI* of cattle exposed to passive strategies declined by 0.04 kg *DMI*/day per unit increase in *THI* and by 0.2 kg *DMI*/day when exposed to active cooling.

## Discussion

Climate change is expected to result in increased frequencies of extreme heat events that will depress crop and pasture production in many areas (Bell et al. 2013; Harrison et al., 2014) and increase the incidence of heat-related animal productivity losses, compromising animal health and welfare (IPCC 2018). While a reduction in *DMI* significantly affects the productivity of cattle during heat events, it is also a reliable indicator to determine the onset of hyperthermia (Polsky and Keyserlingk, 2017). Comparisons of the effects of heat stress across studies have generally been hampered by the fact that past studies of animal heat stress and *DMI* relationships have generally been context specific (e.g. breed or stage of lactation in a dairy cow) and using various heat stress indicators (e.g. ambient temperature and animal body temperatures such as rectal, vaginal or skin surface temperatures), animal respiratory rate and *THI*. In this study, we used a meta-analysis to derive a global relationship between *THI* and *DMI* that accounted for the factors mentioned above. We quantified the relationship between *DMI* and *THI* using only experimental studies, allowing more confidence in the conclusions drawn with respect to differences in subgroups.

### Systematic literature review

The PRISMA framework was effective in the identification and inclusion of suitable studies for this meta-analysis. We obtained a large number of duplicate records (89%; 17,732/19,830), particularly from ISI Web of Science (Fig. 1—Identification). A large proportion of studies were also excluded because these did not focus on relationships between environmental conditions and cattle (95%; 1,676/1,762) (Fig. 1—Screening), which meant that any heat-related variables were not collected. A further 35% of studies (Fig. 1—Eligibility) were excluded even though they investigated heat effects on feed intake in cattle, because of (1) the units reported could not be converted into either *THI* or *DMI* or (2) *THI* and *DMI* datasets could not be paired. This further reinforces the justification for this study; although the literature is rich with data, such information cannot be easily contrasted due to lack of standardisation of dimensions for both heat stress and feed intake.

### Effects of heat stress on DMI

Effects of heat stress have been monitored using many different approaches in the past, from heat chambers to closed barns to modelling. However, few studies differentiate between the effects of acute and chronic heat stress exposure. During heat events, cattle are exposed to hot conditions during the day and, when night-time temperatures are less than around 25 °C, tend to cool during the night by dissipating heat into the environment. However, when night-time conditions are above 25 °C, livestock temperatures and respiratory rates become elevated (Garner et al. 2017). Elevated day-time heat conditions are ameliorated when temperatures fall at night thus reducing milk production losses (Correa-Calderon et al. 2004; Silanikove et al. 2009). However, using the *THI* as a thermal indicator, critical values for *THI*_*min*_ range between 55 (Holter et al. 1997, 1996) and 64 (Igono et al. 1992) depending on cow breeds and regional variability. As such, warm nights contribute to chronic exposure of cattle to heat events, and this further diminishes their capacity to thermoregulate. For this reason, in our study and consistent with previous work from Holter et al. (1996), *THI*_*min*_ is strongly correlated with heat stress (Fig. 2). We found strong negative correlations between THI and *THI*_*min*_ with *DMI* (*r* =  − 0.8), while *THI*_*max*_ was less well correlated (*r* =  − 0.6). This is consistent with previous work that has highlighted the importance of *THI*_*min*_ and night-time temperatures in amelioration of heat stress (Correa-Calderon et al. 2004; Silanikove et al. 2009) and the potential of using *THI*_*min*_ to better assess heat stress in cattle (Holter et al. 1996). However, at present, while *THI*_*min*_ has good potential for estimation of chronic heat stress, daily mean *THI* may be the best measurement for the overall assessment of heat conditions in cattle (day and night).

Regardless of time of day, cattle with elevated core body temperature spend more time standing than lying compared with thermoneutral cattle (Allen et al. 2015). Cattle also show a preference for eating during cooler periods or at night when day-time heat conditions are above optimal (Mallonee et al. 1985; Schneider et al. 1988). These responses are consistent with amelioration of internal heat load during the hottest part of the day (Aharoni et al. 2005; Ominski et al. 2002b). While standing is likely to reduce accumulated internal heat load due to increased skin surface area, reducing feed intake will reduce core body temperature by suppressing heat originating from feed fermentation. A significant research gap relates to the ability accurately predict voluntary feed intake and its potential constraints over a range of scenarios, including heat stress. While mechanistic models have investigated the thermal balance of cattle (Thompson et al. 2014, 2011), no currently available mechanistic animal models have the capacity to capture the biological controls of feed intake in cattle, let alone incorporating the metabolic and physical regulations that occur in heat-stressed cattle. Similarly, while whole farm models such as DairyMod (Johnson et al. 2008) or APSIM (Holzworth, 2014) account for the effects of heat stress on plants, at the time of writing they do not account for animal heat stress or predict the effects of heat on voluntary feed intake in a holistic sense, thereby allowing for interactions between plants and animals.

In contrast to ruminants under thermoneutral conditions, dairy cattle already have an elevated heat load due to higher productivity (Chebel et al. 2004). Differences in heat tolerance exist between cattle breeds, where commonly used dairy breeds (e.g. Holstein–Friesian) are found to more susceptible than beef breeds (Blackshaw and Blackshaw 1994). Here, we did not differentiate between breeds as most were Holstein-Friesians (94.5%). As well, the majority of studies (95%) did not differentiate between crossbreds and purebreds. These findings reveal both the relevance of this study for the dairy sector and emphasise the need to differentiate between heat stress impacts on different breeds. Genetically selecting cows for heat tolerance has been shown to significantly improve heat tolerance of high-yielding dairy cows. Genetic markers to predict heat tolerance in dairy cows were effective at maintaining *DMI* under heat exposure as measured by rectal and vaginal temperature (Garner, 2016). Genomic selection for improved heat tolerance is an adaptation strategy that would be expected to have large benefits, particularly in pasture-based grazing systems where animals are often exposed to ambient conditions. Such an adaptation strategy to future climates would enable better animal welfare outcomes without compromising productivity.

### Effects of heat stress during different lactation phases

We found larger reductions in *DMI* for rising *THI* in early lactation cows than the average population. Reduced *DMI* in early lactation is not only likely to reduce productivity in the short term but may also have implications in longer term if the effect of heat stress is sustained enough to also affect the body reserves and body weight of the cow. In a normal lactation curve, milk production peaks during early lactation by depleting body reserves for milk production (Moran 2005). However, heat-stressed cows have lower ability to mobilise stored peripheral adipose tissue (Baumgard and Rhoads 2012), which further reduces milk production. Lowered *DMI* over a longer period causes gradual depletion of body reserves. Effects of heat stress on productivity and metabolic performance may last beyond the lactation period and into subsequent lactation years, and even have effects on dairy cow offspring (Ouellet et al. 2020). Heat stress exposure in transition cows also reduces milk production (Tao et al. 2019) by altering nutrient metabolism and udder tissue development, potentially resulting in the reduced performance of both dam and calf (Ouellet et al. 2020).

### Effects of animal cooling interventions

Cooling interventions had significant capacity to reduce the impacts of heat stress on *DMI* (Fig. 4), with a divergence in *DMI* between cooled and normal cows observed from a *THI* of 68 onwards. It is already known that the threshold for hyperthermia has significant regional variations due to differences in the local climate, genetic tolerance of cows to heat stress, feed composition and management strategies among others (Nidumolu et al. 2010). For example, *THI* thresholds around the world for dairy cattle vary: in Australia it is set at *THI* 72, in the UK at *THI* 68 and in the USA at *THI* 69. Thus, a global *THI* threshold of 68 is consistent with a potential level at which differences might emerge.

Past work has shown that cooling strategies can offset production losses (Armstrong 1994; Valtorta and Gallardo 2004). In this study, we classified cooling strategies into two main categories: active cooling including the use of sprinklers and fans, and passive cooling for shading (we also categorised a null category as a control treatment). Consistent with previous work, our results showed that both active and passive strategies were effective at reducing heat stress effects on *DMI* (Jones and Hennessy 2000; Nidumolu et al. 2010). However, we found that passive strategies were more effective than active strategies, also consistent with previous work highlighting shading to be more effective that spraying (Jones and Hennessy 2000). In pasture-based systems, shading is also more effective and practical method for cooling cows. Outdoor cattle are more vulnerable to heat stress due to their exposure to ambient conditions, but the application of active methods for cooling are mostly limited to milking times, after which point cattle may have already been exposed to hot conditions. The most effective shading available in outdoor systems is tree shading. Trees also provide other benefits, from habitat for biodiversity, to wind breaks and woody biomass carbon sequestration. In contrast to active cooling methods that require power and water, natural forms of shading such as trees are also relatively inexpensive.

### Implications of DMI variability on milk production

For situations in which no interventions are taken, milk production losses of up to 40% during single heat events are not unusual (West 2003). Although not all such losses can be attributed to *DMI*, lower intake is often the primary factor responsible for reduced lactation in dairy cows (Gao et al., 2017; Wheelock et al. 2010b). A combination of compensatory mechanisms to support the return to thermoneutrality, including shifts in energy demands and nutrient partitioning, is suggested to be responsible for the remaining production losses (Cowley et al. 2015; Shwartz et al. 2009). Further research is required to better understand these processes and quantify their relative contributions to production losses. In this study, we did not investigate the link between reduced *DMI*, *THI* and milk production due to the low number of studies reporting these variables, suggesting that there is a need for more studies studying the nexus of *DMI*, *THI* and milk production.

Reduced *DMI* also reduces milk protein content (Emery 1978; Knapp and Grummer 1991; Rodriquez et al. 1985). A reduction of 29% in *DMI* due to heat stress was reportedly associated with a decline of 33% of milk production and 7% protein content (Shwartz et al. 2009). Protein content in milk is generally the most affected variable during heat events (Chang-Fung-Martel et al., in review) due to varying levels of major nutrients combined with increased demands in extramammary amino acids, resulting in the reprioritisation of amino acids away from milk protein synthesis (Gao et al., 2017). Fat content is affected to a lesser degree and is associated with reduced fibre intake and a shift in the metabolism of carbohydrates, evidenced by increased insulin concentrations and reduced lipolytic stimuli (Baumgard and Rhoads 2012). Effects of heat stress on milk composition may be reduced when cows are fed a total mixed ration and concentrate (Beede and Collier 1986; Bouraoui et al. 2002), highlighting the importance of rationing diets for cows in preparation for heat events. Adjusting diet composition is an effective strategy to ameliorate the impact of heat stress by providing cows with high-quality feeds that deliver appropriate nutrients while reducing heat load from fermentation to improve the performance of cattle in varying climates, but particularly during hot weather.

## Conclusions

This study demonstrated a strong negative correlation (*r* =  − 0.82) between *THI* and *DMI*, suggesting that across continents and stages of lactation, *DMI* was reduced by 0.45 kg/day for every unit in *THI* unit increase. This result allows standardisation of heat stress and feed intake comparison across studies and could be simply applied in whole of farm systems models to improve simulation of the interactions between plants and animals under extreme heat events. Primiparous and multiparous cows did not experience significant differences in the reduction of *DMI* at increasing *THI*s. While differences in the *THI*-*DMI* relationship between lactating and non-lactating cows were not significant, effects of *THI* on *DMI* were significantly different across lactation stages. Passive cooling (e.g. shading) was more effective than active strategies (e.g. fans and sprinklers) at alleviating the reduction in *DMI* at high *THI*s evidenced by lower effects of high *THI* on *DMI*. A divergence in *DMI* between cooled and normal cows observed from a *THI* of 68 onwards indicates that this value could be viewed as a threshold for which cooling interventions could be made.

While reduced *DMI* alone is not sufficient to determine the overall effect in milk production losses in heat-stressed dairy cows, a good understanding of the interactions between *DMI* and *THI* is fundamental to design effective adaptation strategies. Based on our analysis, we recommend an animal-focussed approach that can result in multiple benefits such as reducing yield losses and costs, improving animal welfare and even add to biodiversity outcomes (e.g. trees as shelters from heat stress).
